# Neonatal White Matter Maturation Is Associated With Infant Language Development

**DOI:** 10.3389/fnhum.2019.00434

**Published:** 2019-12-17

**Authors:** Georgina M. Sket, Judith Overfeld, Martin Styner, John H. Gilmore, Sonja Entringer, Pathik D. Wadhwa, Jerod M. Rasmussen, Claudia Buss

**Affiliations:** ^1^Department of Medical Psychology, Berlin Institute of Health, Charité – Universitätsmedizin Berlin, Corporate Member of Freie Universität Berlin, Humboldt-Universität zu Berlin, Berlin, Germany; ^2^Department of Psychiatry, University of North Carolina at Chapel Hill, Chapel Hill, NC, United States; ^3^Development, Health, and Disease Research Program, University of California, Irvine, Orange, CA, United States

**Keywords:** white matter development, infant language development, diffusion tensor imaging, receptive language development, neonatal neuroimaging

## Abstract

**Background:**

While neonates have no sophisticated language skills, the neural basis for acquiring this function is assumed to already be present at birth. Receptive language is measurable by 6 months of age and meaningful speech production by 10–18 months of age. Fiber tracts supporting language processing include the corpus callosum (CC), which plays a key role in the hemispheric lateralization of language; the left arcuate fasciculus (AF), which is associated with syntactic processing; and the right AF, which plays a role in prosody and semantics. We examined if neonatal maturation of these fiber tracts is associated with receptive language development at 12 months of age.

**Methods:**

Diffusion-weighted imaging (DWI) was performed in 86 infants at 26.6 ± 12.2 days post-birth. Receptive language was assessed *via* the MacArthur-Bates Communicative Development Inventory at 12 months of age. Tract-based fractional anisotropy (FA) was determined using the NA-MIC atlas-based fiber analysis toolkit. Associations between neonatal regional FA, adjusted for gestational age at birth and age at scan, and language development at 12 months of age were tested using ANOVA models.

**Results:**

After multiple comparisons correction, higher neonatal FA was positively associated with receptive language at 12 months of age within the genu (*p* < 0.001), rostrum (*p* < 0.001), and tapetum (*p* < 0.001) of the CC and the left fronto-parietal AF (*p* = 0.008). No significant clusters were found in the right AF.

**Conclusion:**

Microstructural development of the CC and the AF in the newborn is associated with receptive language at 12 months of age, demonstrating that interindividual variation in white matter microstructure is relevant for later language development, and indicating that the neural foundation for language processing is laid well ahead of the majority of language acquisition. This suggests that some origins of impaired language development may lie in the intrauterine and potentially neonatal period of life. Understanding how interindividual differences in neonatal brain maturity relate to the acquisition of function, particularly during early development when the brain is in an unparalleled window of plasticity, is key to identifying opportunities for harnessing neuroplasticity in health and disease.

## Introduction

Among unique human abilities, language may be one of the most critical prerequisites for participation in social interaction, and consequently, its acquisition is one of the most important elements of early development. It is one of the earliest functions to develop with large interindividual variability, and well-developed early language skills predict later development of complex cognitions such as theory of mind development (Astington and Jenkins, 1999) and reading achievement (Shapiro et al., 1990). Conversely, early language deficits are associated with various pathologies and neurodevelopmental disorders, including reduced later language abilities (Kuhl et al., 2005), dyslexia (Torppa et al., 2010), autism (Charman et al., 2003), and childhood-onset schizophrenia (Alaghband-Rad et al., 1995). Importantly, language deficits may even precede diagnosis of such disorders, such as the presence of pre-psychotic language difficulties in people with childhood-onset schizophrenia (Alaghband-Rad et al., 1995) and delays in receptive language development at 12 months of age in children later diagnosed with autism spectrum disorder (ASD; Mitchell et al., 2006). More broadly, early language impairment may also impair long-term social adaptation and academic success (Donahue, 1986; Benasich et al., 1993). While neonates have no sophisticated language skills, the neural basis for acquiring this function is assumed to already be present at birth and characterizing relevant neurophenotyes will allow for studying antecedents of this essential ability.

The trajectory of language development has been extensively characterized. The development of receptive and expressive language ability follows universal stages that appear consistent across cultures. Expressive language ability in infants progresses universally from cooing (1–4 months), to babbling (5–10 months), to meaningful speech (10–18 months) (Stoel-Gammon, 1992). The rate of receptive and expressive language acquisition is fastest between 18 and 60 months of age, and slows down after this point (O’Muircheartaigh et al., 2013). Receptive language development is measurable well before expressive language development. Speech discrimination, an aspect of receptive language, has been shown to be predictive of later overall language development by 6 months (Tsao et al., 2004). Children develop from a limited receptive vocabulary of up to 50 words at 12 months of age to a vocabulary of about 5,000 words by around 6 years of age (Locke, 1997). This study focuses on measures of receptive language development in a healthy, normally developing population because receptive language development is already measurable with significant interindividual differences early in infancy and predicts later language development (Tsao et al., 2004).

Normal cognitive development in infants follows an established temporal sequence, and this temporal sequence is assumed to be correlated with the structural maturation of underlying functional brain networks (Dubois et al., 2008). The development of cognitive functions has been linked to maturation (e.g., the onset of myelination) in brain fiber tracts responsible for these functions (Thomason and Thompson, 2011). Previous studies in healthy, normally developing populations have found that language performance in children is related to white matter maturation, and that, for example, the phase of rapid vocabulary development from 18 months onward is underpinned by a concomitant rapid myelination phase in brain regions supporting language, specifically temporal and frontal regions in the left hemisphere (Pujol et al., 2006; Cheng et al., 2018). Historically, much focus has been on differences in regional and overall brain morphology, and the functional variations resulting from such differences. However, *how* brain regions are connected with each other may be at least as important as the anatomical integrity itself in elucidating normal and atypical brain function (Matthews and Fair, 2015). Generally, fibers are known to develop in an asynchronous non-linear spatiotemporal manner, which is assumed to be relevant for the hierarchy of connections between cortical areas. For example, receptive sensory areas responsible for low-level processing tend to mature more quickly than integrative areas responsible for higher-level processing. This is thought to ensure the stabilization of (sensory) information used by the integrative areas for higher-level processing (Qiu et al., 2015).

This study focuses on two specific fiber tracts implicated in language processing, the corpus callosum (CC) and arcuate fasciculus (AF). These fibers vary in respect to their fiber type and developmental time frames. The CC is the main commissural fiber responsible for communication between the two brain hemispheres. The first fibers of the CC can be detected at 10–11 weeks of gestational age (Bloom and Hynd, 2005), and the CC is formed and recognizable at around 18–20 weeks of gestation (Qiu et al., 2015). The number of fibers within the CC is fixed at birth, but changes in the form of increased myelination, redirection, and pruning continue to occur throughout development (Luders et al., 2010). The AF is an association fiber responsible for within-hemispheric cortico-cortical connections that connects the frontal, parietal, and temporal lobes (Catani and De Schotten, 2008). The AF is relatively immature at birth, but has a fast maturation rate in the first year of life when compared with other tracts, for example, those in the motor or sensory domains (Geng et al., 2012). The maturation of the superior longitudinal fasciculus–AF complex, which connects the inferior frontal gyrus with the superior temporal gyrus/sulci and the inferior parietal lobule, has been shown to correlate with the ability to comprehend complex sentence structures in children aged 3 to 10 years (Skeide et al., 2015). More recently, Girault et al. (2019) showed that white matter microstructural development in regions including arcuate tracts at the neonatal time point was associated with receptive language development at 1 and 2 years in healthy children. Both the CC and AF play a role in language processing. The CC plays a key role in the hemispheric lateralization of language (Hinkley et al., 2016). The left AF is associated with syntactic processing (Cheng et al., 2018), while the right AF plays a role in visuospatial processing and some aspects of language processing like prosody and semantics (Catani and De Schotten, 2008).

We here focus on the microstructural development of the CC and AF in healthy infants, as indexed by fractional anisotropy (FA), an MRI-based direct measure of the anisotropy of water diffusing in the brain, and thereby an indirect measure of microstructural properties including axonal coherence, oligodendrocyte proliferation, and myelination. Higher FA values are assumed to correspond with increased white matter maturity. A diverse set of studies highlight the association between white matter maturation, as measured by FA, and cognitive function in healthy infants and children (e.g., Short et al., 2013; Peters et al., 2014; Stjerna et al., 2015; Girault et al., 2019). In addition, FA in infancy potentially has predictive value for children at risk for adverse cognitive outcomes later in development. In infants with familial risk for developmental dyslexia aged 5 to 18 months, FA was significantly lower in the left AF, specifically in the central region of the tract. Furthermore, expressive language, which is a predictor of future reading skills, correlated positively with FA in infants with and without increased dyslexia risk (Langer et al., 2017). This current study aims to elucidate whether interindividual differences in FA, as an index of CC and AF maturity, shortly after birth can predict differences in language development at 1 year of age in a healthy, normally developing population. Because previous findings have demonstrated that white matter fiber tracts develop in an asynchronous fashion, it would be expected that white matter maturation only in specific regions within the CC and AF, and not along the entire length of each tract, would be predictive of later outcomes. Therefore, this study will evaluate FA within the AF and CC in order to examine spatial specificity within the tracts that may be particularly relevant for language outcomes at 12 months of age.

## Materials and Methods

### Sample

The cohort of *N* = 86 infants [43 (55.1%) males] included here is a subsample of children that are part of a prospective longitudinal study of pregnant women and their offspring conducted at the University of California, Irvine, Development, Health, and Disease Research Program (see Table 1). Exclusionary criteria for infants were as follows: birth before 34 weeks of gestation, and evidence of a congenital, genetic, or neurologic disorder. The subsample was selected based on availability of neuroimaging data as newborns (mean gestational age: 38.96 ± 1.39 weeks, mean postnatal age at scan = 26.26 ± 12.25 days) and language developmental outcomes at the age of 12 months (mean age = 11.47 ± 2.02 months). Two infants were born after 34 weeks of gestational age but prior to 36 weeks of gestational age: one at 34 weeks and 4 days, and one at 35 weeks and 4 days; results were unchanged after re-running analyses to exclude the two late preterm births. This subsample can be considered representative of the full sample of children in this cohort because it did not differ from the complete cohort of infants (*N* = 106) in terms of key sociodemographic characteristics (all *p* > 0.05; see Supplementary Table S1). The UCI Institutional Review Board approved all study procedures, and written informed consent was obtained from all mothers.

**TABLE 1:** Demographic data of participants included in the analyses (*N* = 86).

**Population**	**Variable**	**Mean**	**Range**
Mother (*N* = 86)	Age (years)	27.79 ± 5.65	18 to 41
	Maternal IQ	94.37 ± 10.91	69 to 117
	Maternal depression	0.59 ± 0.52	0 to 2.25
	SES+	3.11 ± 0.93	1 to 5
Infant (*N* = 86)	Gestational age (weeks)	38.96 ± 1.391	34 to 41
	Scan Age (days)	26.26 ± 12.246	5 to 58
	Postnatal environment (HOME)	35.04 ± 3.696	23 to 42
	MCDI – PU^∗^	16.91 ± 5.63	6 to 28
	Sex	43 (55.1%) males	35 (44.9%) females
Infant	White non-Hispanic	42.5%	
race/Ethnicity (%)	White Hispanic	28.7%	
	Asian	9.2%	
	Other	19.6%	
Household highest Level of maternal education (%)	High-school or test equivalent	22.4%	
	Vocational school or some college	45.9%	
	Associates degree	3.5%	
	Bachelors or graduate level degree	28.3%	
Gross annual household income (%)	<$15,000	11.1%	
	$15,000–$29,9999	17.3%	
	$30,000–$49,999	22.2%	
	$50,000–$100,000	42.0%	
	>$100,000	7.4%	

*Gestational age is gestational age at birth. Maternal IQ was assessed using subscales of the Wechsler Adult Intelligence Scale; Maternal depressive symptoms were assessed with the Center for Epidemiologic Studies –Depression Scale (CES-D); SES, socioeconomic status; MCDI, MacArthur-Bates communicative development inventory; ^∗^PU, phrases understood. +SES included in analyses consisted of a summary variable including both household income and highest maternal education.*

### Neuroimaging

#### Diffusion-Weighted Imaging

Diffusion-weighted imaging (DWI) data were acquired during natural sleep using a 12-channel head receive coil at 3T field strength on a Siemens TIM Trio system in 106 neonates. After feeding and soothing to the point of sleep, neonates were placed in a CIVCO beaded pillow^1^. The pillow covered the neonates’ body and head, became rigid under vacuum, and provided a comforting swaddle, motion prevention, and hearing protection when used in conjunction with standard foam earplugs. A pediatric specialist observed participants throughout the duration of the scans, monitoring for heart rate and oxygen saturation *via* a pulse oximeter attached to the foot. The 49-direction diffusion protocol (EPI; repetition time/echo time = 8900/83 ms, matrix = 256 × 256 × 75, resolution = 2 mm× 2 mm× 2 mm, 42 unique directions at *b*-1,000 s/mm^2^, 7 at *b* = 0) was 7 min and 43 s long.

#### Fiber Profile Generation

The diffusion profile measurements were generated using the NA-MIC atlas-based fiber analysis toolkit (Verde et al., 2014). In brief, diffusion datasets were first rigorously checked for appropriate quality (Oguz et al., 2014), which included slice-wise and gradient-wise artifact detection (outlying gradients were censored from analysis), as well as eddy current, motion correction, and b-vector rotation. Eddy current, motion correction, and b-vector rotation were performed *via* a tool called DTIPrep (Liu et al., 2010). Baseline averaging was performed *via* rigid registration to an iteratively improving average baseline (see Oguz et al., 2014). Upon completion of the automatic quality control (QC), visual QC was performed.

Participants (*n* = 20) were removed from analysis if they had less than 28 of 42 remaining good directions after the initial QC process to ensure similar signal-to-noise ratio. Data quality impairments were the result of motion artifacts. Remaining participants had bad directions censored prior to the derivation of diffusion metrics (e.g., FA). Participants included in analysis had 35.6 ± 4.3 (SD) remaining gradients on average, with a range of 28–42 included gradients. This was followed by weighted least square tensor estimation, skull stripping *via* prior brain mask from co-registered structural T2-weighted images, and unbiased study-specific diffusion tensor imaging (DTI) atlas building. The sample-based atlas specific to the current study’s acquisition parameters and age range was built using DTIAtlasBuilder^2^ *via* a three-step registration: (1) affine registration, (2) unbiased diffeomorphic atlas computation (Joshi et al., 2004), and (3) refinement with a symmetric diffeomorphic registration *via* Advanced Normalization Tools [ANTS (Pineda et al., 2014)]. Atlas building included the original (*N* = 86) participants with sufficient quality data. Fiber tract streamline DTI tractography was performed *via* 3D Slicer (version 4.3.0)^3^ in the DTI atlas space followed by fiber cleaning with FiberViewerLight. Fiber profiles were sampled at a 1 mm step size along both directions away from the fiber’s intersection with the origin plane (plane that intersects the fiber bundle orthogonally at the median fiber location). Fiber profiles of FA were extracted after fiber parameterization for profile analysis.

### Characterization of Infant Language Development

Infant language development was assessed using the MacArthur-Bates Communicative Development Inventory (MCDI; Fensen et al., 1993) at 12 months of age. The MCDI is a survey for parents that is used to assess language development and has been shown to be a valid measurement in both typically developing children and children with developmental abnormalities. The MCDI’s research applications include screening and preselecting children at different levels of language development (including those with particular language characteristics or unusual profiles), matching children on language skills, and examining the influence of other variables on language development. Evidence of the MCDI’s validity comes in the form of both convergent and concurrent validity. The evidence for convergent validity rests on the fact that developmental functions assessed in the MCDI’s subscales correspond well with those reported in observational studies. In addition, concurrent validity of the MCDI was assessed by comparing performance on various MCDI scales with performance on standardized and laboratory measures of language abilities. The correlations between laboratory measures and MCDI scores were generally substantial in a wide array of studies in normally developing and neurodevelopmentally delayed populations (Fenson et al., 2007). For the purposes of analyses, the “Phrases Understood” (PU) score from the MCDI: Words and Gestures form was selected. The PU score shows the number of common phrases reported as understood (e.g., “Give me a hug,” “Stop it,” and “Come here”), and was selected because of the study’s focus on receptive language, which develops earlier than expressive language, and substantial interindividual variability can be expected at 12 months of age. The PU score gives a snapshot of more sophisticated language development going beyond single word familiarity/recognition (“Words Understood” score from MCDI), because the phrases communicate real-world actions and situations, require the infants to process contextual cues as well as possibly sounds and gestures accompanying the phrases, and are indicative of participation in social communication and interaction, the telos of language skill.

### Analytic Approach

Tract-based profile analysis considered the association of FA values at each individual point along the CC and AF with language development at 12 months of age using a MATLAB ANOVA model. The same analyses were conducted using the left and right fornix as a control tract. Clusters significantly associated with receptive language after considering the multiple comparisons made along the length of the CC and AF were identified using a Monte Carlo simulation approach with 10,000 iterations to identify necessary cluster properties (extent and effect size) for significance at a corrected threshold of *p* < 0.05. FA values for each point along the fiber tract were corrected for gestational age at birth and postnatal age of the neonate at the time of the DWI scan within the ANOVA model. Associated clusters were defined as >10 consecutive significant points (*p* < 0.05). In a second step, we tested in SPSS 23.0 whether additional potentially confounding variables were associated with the peak cluster FA values identified in the first analysis step using correlation analyses as a basis for determining whether these variables needed to be included in the main model; these variables included infant sex, maternal socio-economic status (SES), quality of the infants’ home environment, maternal IQ, and maternal depressive symptoms (see below; Table 1 and Supplementary Table S2). FA clusters associated with of language development were then plotted and visualized using MATLAB.

Additional analyses were conducted to examine if FA across entire tracts was associated with language development. FA across the entire tract was averaged, and the average value was correlated with language development at 12 months.

### Covariates

Gestational age and age at DWI scan were controlled for in all analyses to adjust for time spent in and *ex utero* at the time of the MRI assessment because there is inter-individual variability in these variables that are significantly associated with tract maturity (van Kooij et al., 2012; Dubois et al., 2014; Rasmussen et al., 2017). Infant sex was examined because there is evidence for sex differences in the maturation of DTI diffusion indices (e.g., Szeszko et al., 2003; Westerhausen et al., 2004; Rose et al., 2009) as well as in language development (e.g., Huttenlocher et al., 1991). Furthermore, correlation analyses were conducted for SES because of its impact on cognitive development (Wong and Edwards, 2013) and because SES has been found to occasionally lead to underreporting of child performance on surveys completed by parents (Fenson et al., 2007). Maternal SES was assessed using a summary variable consisting of overall household income as well as highest level of maternal education obtained. The quality of the infants’ early home environment has been shown to play an important role in early cognitive development (e.g., Totsika and Sylva, 2004), and thus, this was tested for using the Infant-Toddler Home Observation for Measurement of the Environment Inventory (Caldwell and Bradley, 1984), which is based on home observations and semi-structured interviews when infants were 6 months of age. The Infant-Toddler Home Observation for Measurement of the Environment Inventory gives an estimate of the quality of the caregiving environment based on a composite score consisting of parental responsivity, parental involvement, acceptance of the child, access to a variety in daily stimulation, the provision of appropriate play materials, and involvement in activities that afford learning. Maternal IQ is associated with her child’s cognitive development and was therefore also examined as a potential covariate in the analyses. Maternal intellectual ability was assessed using the Perceptual Reasoning Index (consisting of block design, matrix reasoning, and visual puzzles) from the Wechsler Adult Intelligence Scale (Wechsler, 1955). Finally, maternal depressive symptoms were included in the correlation analyses because maternal mood has been shown to alter the mother’s perception of her infant’s behavior and may thus affect her report of her child’s language developmental status (McGrath et al., 2008). Also, maternal depression has been shown to negatively correlate with infant expressive communication (Kaplan et al., 2014) and cognition (Azak, 2012). Maternal depressive symptoms were assessed with the Center for Epidemiologic Studies – Depression Scale (Radloff, 1977). Correlation analyses found that none of the confounding factors were significantly associated with the outcomes of interest (*p* < 0.05), suggesting that it is appropriate to exclude them from the primary analyses (see Supplementary Table S2).

## Results

### Receptive Language

The mean number of PU in our sample cohort was 16.9 ± 5.6 showing that our study population’s scores were generally in line with expected values (published MCDI norms at 12 months of age = 15.8 ± 5.5; Fenson et al., 2007). *N* = 8 infants were excluded from analyses due to lack of language scores.

### Neonatal FA Along the CC Is Associated With Receptive Language at 12 Months of Age

**FIGURE 1:**
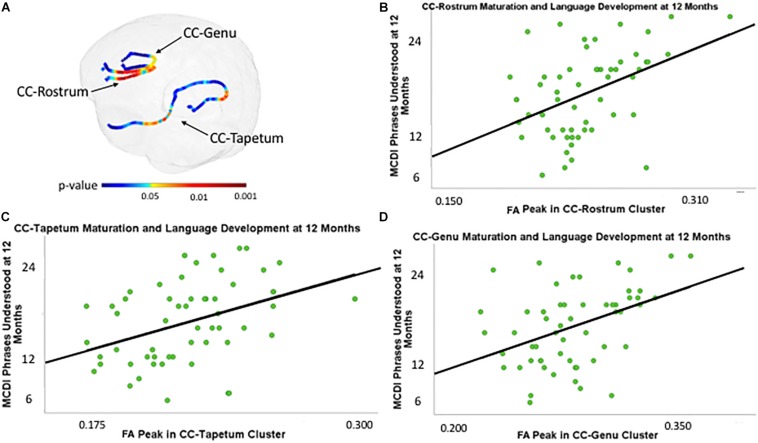
**(A)** Clusters within the genu, rostrum, and tapetum of the corpus callosum associated with MacArthur-Bates Communicative Development Inventory (MCDI)-Phrases Understood (PU) at 12 months of age controlling for gestational age at birth and age at scan, and PU at 12 months of age plotted against fractional anisotropy (FA) values (scalar values between 0 and 1, with 1 representing unidirectional movement) of the point with the highest *p*-value within clusters in the rostrum **(B)**, tapetum **(C)**, and genu **(D)** of the corpus callosum (CC) controlling for gestational age at birth and age at scan.

There were three regions within the CC with clusters that were associated with 12-month PU scores at the corrected *p* < 0.05 level. These clusters were located within the genu, rostrum, and tapetum (Figure 1). Table 2 summarizes the results and provides information on cluster size, percentage of tract significantly correlated with PU, corrected peak significance, and beta values.

**TABLE 2:** Clusters associated with 12-month MCDI “PU” scores after adjustment for gestational age at birth and postnatal age of the neonate at the time of the DWI scan.

**Cluster**	**Cluster size**	**Percentage of tract**	**Peak significance (corrected)**	**Beta**
CC genu	42 points	48.8%	pMC = 0.001	101.814
CC rostrum	42 points	47.7%	pMC < 0.001	113.92
CC tapetum	21 points	11.7%	pMC = 0.019	112.6
Left fronto-parietal AF	90 points	15.6%	pMC = 0.008	94.129

*Cluster size is indicated by the number of consecutive points in that cluster significantly associated with language outcomes; percentage of tract represents the overall percentage of tract represented by cluster; corrected peak significance in cluster; and beta value at peak significance. AF, arcuate fasciculus; CC, corpus callosum; DWI, diffusion-weighted imaging; MCDI, MacArthur-Bates communicative development inventory.*

### Neonatal FA Along the AF Is Associated With Receptive Language at 12 Months of Age

**FIGURE 2:**
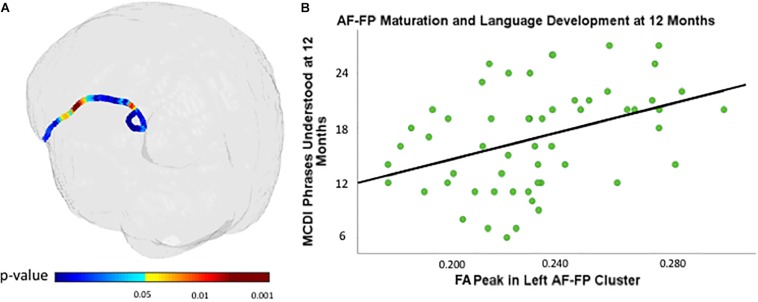
Cluster within the fronto-parietal region of the left arcuate fasciculus (AF) associated with MacArthur-Bates Communicative Development Inventory (MCDI)-PU at 12 months of age controlling for gestational age at birth and age at scan **(A)** and PU at 12 months of age plotted against neonatal FA values (scalar values between 0 and 1, with 1 representing unidirectional movement) of most significantly associated points within the cluster in the AF **(B)** controlling for gestational age at birth and age at scan.

A cluster within the fronto-parietal region of the left AF is associated with 12-month PU scores (see Figure 2 and Table 2 for details). There were no clusters within the right AF that were significantly associated with PU.

### Neonatal FA in the Left and Right Fornix Is Not Associated With Language Development

Additional analyses were conducted to see if associations could be found between language development and the right and left fornix, tracts known to be associated with episodic memory (Douet and Chang, 2015) but not thought to be implicated in language development. No significant association was found between clusters and language development in the left and right fornix (see Supplementary Figure S1).

### Mean FA

Mean FA across the AF and fornix is not associated with language development at 12 months (see Table 3). However, mean FA in the tapetum, rostrum, and genu of the CC is associated with language development at 12 months (*p* < 0.05).

**TABLE 3:** Mean fractional anisotropy (FA) across fiber tracts and their association with MacArthur-Bates Communicative Development Inventory (MCDI)-PU at 12 months of age.

	**Average FA**	**Pearson correlation**	**p-statistic**
MCDI – Phrases	Left AF-FP	0.135	0.320
understood	Right AF-FP	0.149	0.273
	CC Tapetum	0.331	0.013^∗^
	CC Rostrum	0.312	0.019^∗^
	CC Genu	0.279	0.038^∗^
	Left fornix	0.221	0.102
	Right fornix	0.251	0.062

*^∗^Correlation significant at *p* = 0.05.*

## Discussion

Remarkably, already at the neonatal time point, there were three regions within the CC and one region within the left AF in which FA, a robust global index of white matter microstructural development, was positively correlated with receptive language development at 12 months of age. These findings are in line with observations in infants, older children, and adults that suggest a relationship between higher white matter maturation in brain structures, including CC and AF, as assessed by FA, and higher cognitive function, and suggest that this relationship exists already before most language development is measurable. In addition, this study was able to reproduce prior findings suggesting that higher FA in the left AF in particular was positively associated with language development (Lebel and Beaulieu, 2009), both validating the importance of the role of AF in receptive language and suggesting that regional lateralization effects related to language typically found later in childhood are already present in healthy neonates.

Importantly, this study was able to demonstrate that interindividual variation in FA in tracts associated specifically with language function (the CC and left AF) already in neonates is meaningfully associated with language acquisition, despite the fact that major features of this ability are largely absent at birth. This is in line with recent findings by Girault et al. (2019), who found that white matter microstructural development in tracts including the AF in newborns was associated with cognitive development as assessed using the Mullen Scales of Early Learning generally, as well as language development as assessed by Mullen Scales of Early Learning subscales specifically. Girault and colleagues chiefly found associations between infant cognitive outcomes and neonatal white matter maturation as assessed by axial diffusivity, a DWI measure assessing longitudinal diffusivity along the tensor ellipsoid main axis. During white matter maturation, axial diffusivity generally decreases as FA increases, and thus, these findings are commensurate. In addition, while Girault and colleagues did not find neonatal FA to be associated with 1-year receptive language outcomes, they determined that neonatal FA specifically in the left fronto-parietal and fronto-temporal AF was associated with receptive language development at 2 years, conserving laterality, regional specificity, and effect direction across the studies. Thus, we conclude that this relationship between neonatal white matter maturity generalizes both across samples and varying instruments to assess language development. Using a tract not thought to be associated with language development (left and right fornix), this relationship was not found. The fact that the microstructural development of the CC and AF at birth is already associated with later language development supports the idea that the foundation of language processing is laid well in advance of the majority of language development and the emergence of measurable language skills. This notion is supported by the observation that in infants born at 28–32 weeks of gestation age and tested 3 days after birth, a phoneme-sensitive cortical network was already shown to be functional based on discrimination responses to a change of phoneme (Mahmoudzadeh et al., 2013).

The findings of the current study suggest that interindividual variability in maturation of white matter fiber tracts lays an important foundation for acquiring later cognitive skills. An infant’s acquisition of language skills is largely influenced by the level and types of exposure to language in its postnatal caregiving environment. Underpinning this relationship is a concomitant growth and maturation of brain language pathways, which is contingent on exposure to language. It is known that this maturation can be severely affected by deprivation of expected influences in select critical or sensitive periods. Cheng and colleagues, for example, showed reduced connectivity in language-relevant white matter pathways, including lower FA in the left AF, of deaf children who lacked early language exposure. Such microstructural alterations were accompanied by specific deficits including reduced comprehension of sentence structure (Cheng et al., 2018). Conversely, Pujol et al. (2006) found that accelerated vocabulary development after 18 months is related to a rapid myelination phase in language-related temporal and frontal brain regions. Given the positive association between brain maturation and increasingly sophisticated language processing, it is likely that early differences in white matter microstructure also influence how language skills themselves are acquired. Thus, differences in white matter microstructure may already be associated with later language development in part because they affect the ability to learn language.

Given that neonatal white matter microstructure is associated with later language development, it becomes particularly pertinent to determine the origins of white matter development at the neonatal time point. Due to the fact that brain MRI scans were conducted shortly after birth, differences in the infants’ postnatal caregiving environments and exposure to language are unlikely to account for the variation in fiber tract maturation observed here. Interestingly, the prenatal environment has been shown to affect language development. Exposure to not only cocaine (Morrow et al., 2003), alcohol (Greene et al., 1990), and mercury (Crump et al., 1998; Lewis et al., 2011) but also maternal stress has been linked to lower overall language skills in toddlers and children, with prenatal stress linked specifically to reduced receptive language abilities in toddlers (Laplante et al., 2004). These environmental variables have the potential, *via* placental transfer of these teratogens or stress-sensitive biological mediators, to alter the trajectory of the developing fetal brain (Moog et al., 2017; Arck et al., 2018; Graham et al., 2018; Heim et al., 2018; Rasmussen et al., 2018; Rudolph et al., 2018).

This study found that specific, defined FA clusters within each fiber tract, rather than global FA across the entire tract was highly associated with language development at the age of 12 months. Additional analyses demonstrate that mean FA across the tapetum, rostrum, and genu of the CC is associated with language development at 12 months, but no such relationship can be established within the left AF. This strengthens the argument for the importance of the asynchronous development within tracts. Given the size of the clusters within the CC tracts and the strength of the association with the outcome variable, it would be expected that overall FA would also have some relationship with language development. However, the association for FA across the tract is much weaker than that for FA within the specific clusters. Mean FA within the left AF has no association with language outcomes, and this can be explained by the relatively small size of the associated cluster within the structure. These findings are in line with previous work (e.g., Yakovlev and Lecours, 1967; Brody et al., 1987; Dubois et al., 2008) establishing that white matter develops in an asynchronous spatiotemporal fashion. This non-linear relationship is thought to be significant for information processing, with areas responsible for lower-level processing maturing generally more quickly in order to ensure the stabilization of information and areas responsible for higher-level processing generally maturing later, and the findings in this study are reasonable in the context of the hierarchy of information processing. The CC develops early in gestation and is relatively mature at birth, and the hemispheric lateralization of language processing – a function of the CC – is a basic function relevant to language development. Therefore, given its more basic function, it is reasonable to expect that large areas within the CC are highly relevant for language development and that earlier, more advanced functionality in these specific regions would be robustly associated with later language phenotype. While little is known about the functional specificity of microstructural variation within the CC, evidence suggests that the CC follows an anterior-to-posterior maturation gradient, potentially accounting for the relative importance of the genu, rostrum, and tapetum at the neonatal time point, as all three are located within the anterior third of the CC (Luders et al., 2010). The AF develops later in gestation, is less mature at birth, and is implicated in syntactic processing, a reasonably advanced form of language processing. The portion of the tract associated with 12-month language development outcomes at the neonatal time point is relatively smaller in the AF compared to the CC, which fits within the established framework of areas responsible for higher-order processing developing later or being slower to mature. However, this study was not only able to identify a region within the AF associated with language outcomes but also able to do so specifically within a region of the left AF. This regional lateralization effect is in line with observations in older children that suggest that more basic language processing functions within the AF are left-lateralized, while the right AF plays a role in much more advanced forms of language processing, such as prosody and semantics, which is why it is expected to not find associated clusters within the right AF at the neonatal time point.

The present work has some limitations. First, single-shelled low *b*-value data collection restricted analysis to the standard Diffusion Tensor model as opposed to multi-shelled high angular resolution approaches that enable more nuanced insight into microstructural makeup. Second, data collection did not employ a reverse phase encoding scheme, meaning that distortion correction was not performed in the current study. By nature, acquisition of infant DWI data is difficult, and motion occurring during natural sleep may impair the quality of acquired data and thus lead to data loss. Infant language development was assessed *via* mother-reported questionnaires, which are likely to contain some bias. Statements concerning laterality effects were based on observations in one hemisphere and not the other, but not formally tested using a tract-based lateralization index. In addition, longer follow-up is necessary to determine how stable the relationship between neonatal white matter maturation and language development is. A prospective study evaluating infants at high risk for autism found that infants who developed ASD had fiber tracts characterized by higher FA at 6 months, followed by a slower change over time relative to infants without ASD, leading to lower FA values in those with ASD by 24 months (Wolff et al., 2012). Therefore, future studies should characterize the trajectory of change in white matter maturation from birth to 12 months age and its association with cognitive outcomes. One potential follow-up would be examining longitudinal data from the present cohort and evaluating how change in white matter fiber tracts is associated with later language development.

In summary, we confirm and extend previous findings on the relationship between early postnatal white matter maturation and cognitive development. The study was able to determine that greater white matter maturation at the neonatal time point was positively associated with receptive language development at 12 months of age and confirm the importance of the left AF in language-associated outcomes. Such findings increase the utility of FA as a developmental marker that can be used to evaluate normal or abnormal development in healthy and clinical populations. Understanding how interindividual differences in the neonatal brain relate to the acquisition of function, particularly during early development when the brain is in an unparalleled window of plasticity, is key to identifying opportunities for harnessing neuroplasticity in health and disease to optimize outcomes. In addition, these specific findings indicate that language development, which is a skill that only becomes measurable significantly after birth, is already associated with neonatal brain development. This highlights the fact that language development is not just a function of exposure, but rather is guided even in its earliest stages by brain maturation.

## Data Availability Statement

The datasets generated for this study are available on request to the corresponding author.

## Ethics Statement

The studies involving human participants were reviewed and approved by the University of California, Irvine Institutional Review Board. Written informed consent to participate in this study was provided by the participants and the participants’ legal guardian/next of kin.

## Author Contributions

All authors listed have made a substantial, direct and intellectual contribution to the work, and approved it for publication.

## Conflict of Interest

The authors declare that the research was conducted in the absence of any commercial or financial relationships that could be construed as a potential conflict of interest.

## Footnotes

1 www.civco.com

2 https://www.nitrc.org/projects/dtiatlasbuilder

3 http://www.slicer.org
