# Supplementary material for: Neonatal White Matter Maturation Is Associated With Infant Language Development
Source: Front Hum Neurosci. 2019 Dec 17;13:434. doi: 10.3389/fnhum.2019.00434 (PMC6927985; doi:10.3389/fnhum.2019.00434)
Supplement: Supplementary file 1 [file Data_Sheet_1.PDF]

Population	Variable	t	p
**Mother** (N=86 vs. 106)	Age (years)	-1.068	0.288
	Maternal IQ	0.153	0.879
	Maternal Depression	1.123	0.222
	SES+	-0.861	0.391
**Infant** (N=86 vs. 106)	Gestational Age (weeks)	1.606	0.111
	Scan Age (days)	-0.243	0.809
	Postnatal Environment (HOME)	1.248	0.215
	MCDI -- PU*	0.631	0.530
	Sex	0.028	0.978
**Infant Race/Ethnicity (%)**	White non-Hispanic	-1.007	0.316
	White Hispanic		
	Asian		
	Other		
**Household Highest Level of Maternal Education (%)**	High-School or Test Equivalent	-1.134	0.259
	Vocational School or Some College		
	Associates Degree		
	Bachelors or Graduate Level Degree		
**Gross Annual Household Income (%)**	<$15,000	0.016	0.988
	$15,000-$29,9999		
	$30,000-$49,999		
	$50,000-$100,000		
	>$100,000		

Supplementary Table 1. Results from independent-samples t-test verifying that sample included in analysis can be considered representative of the full sample of children in this cohort because it did not differ from the complete cohort of infants (N=106) in terms of key sociodemographic characteristics (all p>0.05). Gestational age is gestational age at birth. Maternal IQ was assessed using subscales of the Wechsler Adult Intelligence Scale; Maternal depressive symptoms were assessed with the Center for Epidemiologic Studies -- Depression Scale (CES-D); SES = socioeconomic status, MCDI = MacArthur-Bates Communicative Development Inventory; *PU = Phrases Understood. +SES included in analyses consisted of a summary variable including both household income and highest maternal education.

		Pearson Correlation	p-statistic
**Infant sex**	Peak CC Tapetum	0.097	0.372
	Peak CC Rostrum	-0.026	0.813
	Peak CC Genu	-0.022	0.838
	Peak Left Arcuate-FP	0.039	0.0719
	Language Development	-0.049	0.0652
**Maternal IQ**	Peak CC Tapetum	0.080	0.472
	Peak CC Rostrum	-0.033	0.763
	Peak CC Genu	-0.068	0.537
	Peak Left Arcuate-FP	-0.037	0.737
	Language Development	-0.179	0.192
**Maternal Depression**	Peak CC Tapetum	-0.155	0.237
	Peak CC Rostrum	0.02	0.878
	Peak CC Genu	-0.019	0.883
	Peak Left Arcuate-FP	0.129	0.326
	Language Development	-0.074	0.628
**SES**	Peak CC Tapetum	0.00	0.997
	Peak CC Rostrum	-0.029	0.791
	Peak CC Genu	0.012	0.910
	Peak Left Arcuate-FP	0.122	0.264
	Language Development	-0.123	0.367
**Infant postnatal environment**	Peak CC Tapetum	0.176	0.129
	Peak CC Rostrum	0.127	0.274
	Peak CC Genu	0.135	0.244
	Peak Left Arcuate-FP	0.071	0.541
	Language Development	-0.004	0.975

Supplementary Table 2. Correlations between main variables of interest, i.e. peak values in FA clusters associated with language outcomes as well as language development as measured by 12-month MCDI- "Phrases Understood," and potentially confounding variables. None of the confounding factors were significantly associated with the outcomes of interest ($p<0.05$), suggesting it is appropriate to exclude them from the primary analyses.

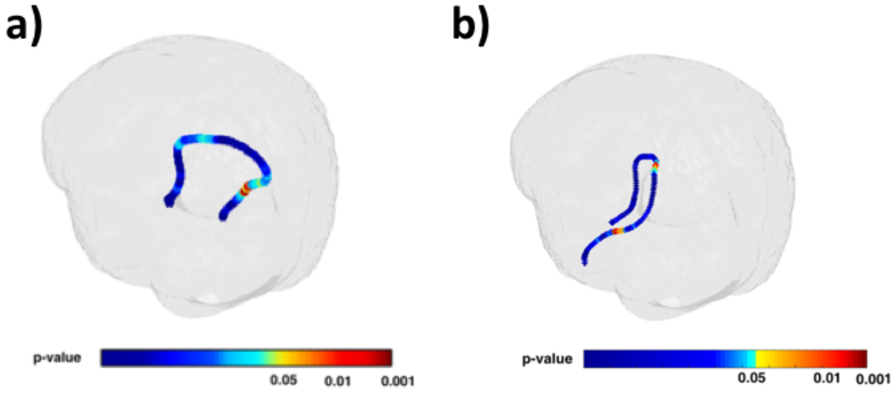

Supplementary Figure 1. The a) right and b) left fornix as control tracts. No FA clusters (>10 points) within the right and left fornix predict MCDI-Phrases Understood at 12 months of age.
